# Totally 3D endoscopic aortic valve replacement: initial results and experience from a single center

**DOI:** 10.3389/fcvm.2024.1468452

**Published:** 2024-10-09

**Authors:** Huu Cong Nguyen, Dat Thanh Pham

**Affiliations:** ^1^ E Hospital, Hanoi, Vietnam; ^2^ University of Medicine and Pharmacy - Vietnam National University, Hanoi, Vietnam; ^3^ Department of Cardiovascular and Thoracic Surgery, Cardiovascular Center, E Hospital, Hanoi, Vietnam

**Keywords:** totally endoscopic, aortic valve replacement, TEAVR, 3D endoscopy, fourth intercostal space, minimally invasive cardiac surgery, aortic stenosis, aortic regurgitation

## Abstract

**Objective:**

This study aimed to evaluate the feasibility and initial outcomes of totally endoscopic aortic valve replacement (TEAVR) performed via a single working port at the fourth intercostal space (ICS) utilizing a 3D endoscopic system.

**Methods:**

A retrospective observational study was conducted on 35 consecutive patients who underwent TEAVR over a six-month period from December 2023 to June 2024. Patient selection was based on the presence of isolated aortic valve disease without the need for ascending aorta replacement or aortic root enlargement. A 4 cm single working port was created at the 4th ICS, extending from the right mid-axillary to the anterior axillary line. A 10-mm trocar for a 3D endoscope was placed at the right anterior-axillary line. Peripheral cardiopulmonary bypass (CPB) was established. The primary outcomes investigated included the success rate of the procedure, in-hospital mortality, and perioperative complications.

**Results:**

The mean age of the patients was 58.7 ± 12.8 years, with 22.9% being female. The majority of patients (77.1%) presented with aortic stenosis, often accompanied by severe calcification. The medianCPB time was 210 ± 43 min, and the median aortic cross-clamp time was 132 ± 41 min. The procedure was successfully completed in all patients using the endoscopic approach, with no conversions to full sternotomy. Two mortalities were recorded, attributed to postoperative complications including bleeding and cerebral infarctions. The early (30-day) mortality rate was 5.7%. Prolonged mechanical ventilation (>48 h) was required in 17.1% of patients, and reoperation for bleeding was necessary in 2.9% of patients.

**Conclusions:**

TEAVR is a feasible procedure with the potential to replace the traditional sternotomy approach for aortic valve replacement.

## 1 Introduction

Over the past two decades, endoscopic cardiac surgery has evolved significantly, offering numerous benefits to patients. These benefits include minimized surgical trauma, reduced postoperative pain, expedited recovery, and shortened hospital stays (1). The development of minimally invasive techniques and technological advancements, particularly the introduction of 3D endoscopic systems, have gained traction across all surgical disciplines, including aortic valve surgery.

There are various approaches in endoscopic or minimally invasive aortic valve replacement. Different surgical approaches are reported, such as partial upper sternotomy, parasternal minithoracotomy, right anterior minithoracotomy, and transaxillary incision (2). Each procedure has its own advantages and disadvantages, as documented by numerous authors globally. Several reports provide practical technical insights, with many indicating that smaller incisions can reduce surgical morbidity (1, 2). Compared to other minimally invasive approaches, TEAVR performed via a single working port offers distinct advantages. The 3D endoscopic system significantly enhances the surgeon's visualization, providing greater precision in the surgical field, which is particularly beneficial in complex cases. Furthermore, the comprehensive application of 3D endoscopy allows surgeons to operate without reliance on direct vision, thereby minimizing the extent of the incision. This reduction in procedural invasiveness facilitates faster recovery, decreases the length of hospital stay, and improves postoperative mobilization for patients.

In this study, we aim to evaluate the feasibility and initial outcomes of TEAVR performed via a single working port at the fourth ICS utilizing a 3D endoscopic system. This study specifically focuses on the success rate of the procedure, early outcomes including in-hospital mortality, and perioperative complications.

## 2 Material and methods

### 2.1 Patient selection and data collection

A retrospective, observational study was conducted using prospectively collected data from 35 consecutive patients who underwent totally endoscopic aortic valve surgery over a six-month period, from December 2023 to June 2024. Routine preoperative evaluations included coronary angiography and computed tomography scans to assess intrathoracic aortic position and peripheral vascular disease.

Inclusion criteria for TEAVR comprised isolated aortic valve disease without the need for ascending aorta replacement or aortic root enlargement. Exclusion criteria included strong pleural adhesions, active endocarditis with abscess involving the aortic annulus, severe aortic atherosclerosis, and inability to achieve peripheral cannulation. Patients in urgent or emergency conditions were also excluded from the study.

The primary outcomes investigated were the success rate of endoscopic aortic valve replacement, the conversion rate to sternotomy (for any reason: uncontrolled bleeding or complex lesions that could not be managed endoscopically, inadequate valve exposure, insufficient CPB flow…) intraoperative complications, in-hospital mortality, perioperative complications, duration of mechanical ventilation, length of intensive care unit (ICU) stay, and blood transfusion rate. This study was approved by the ethics review committee of E Hospital Hanoi, Vietnam, and the University of Medicine and Pharmacy - Vietnam National University, Hanoi.

### 2.2 Surgical technique

**Figure 1:**
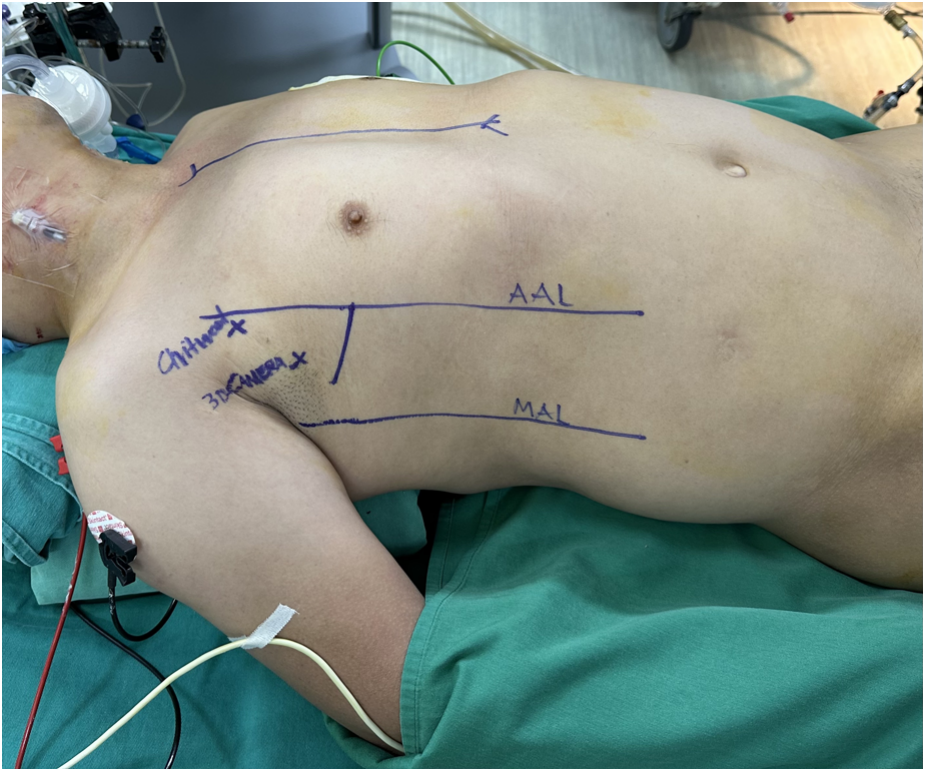
Patient positioning.

The operation commenced under general anesthesia with double-lumen or single-lumen tracheal intubation. The patient was positioned supine with the right side elevated to a 30-degree angle by placing a pillow beneath the axilla. The right upper limb was positioned parallel to the body with the elbow joint slightly flexed to fully expose the right lateral chest (Figure 1).

**Figure 2:**
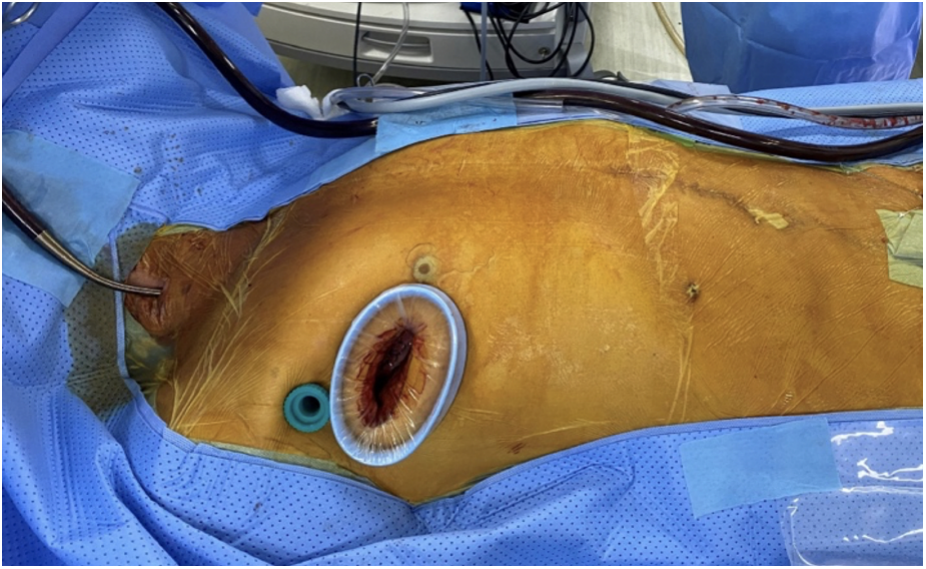
Placement of single working port and 3D endoscopic camera.

Peripheral CPB was established via the right femoral artery and vein. A superior vena cava cannula was placed through the right internal jugular vein. A single working port was created at the 4th ICS with a 4 cm incision extending from the mid-axillary line to the anterior axillary line on the right side. After the incision, a soft tissue retractor was inserted to avoid damage to the ribs and surrounding structures (Figure 2). This single working port was used for all surgical instruments, the cardioplegia needle, the left venting line, and the CO_2_ insufflator.

A 10-mm trocar for a 3D endoscope (Karl Storz, Tuttlingen, Germany) was inserted through the 3rd ICS, positioned at either the right mid-axillary line or the right anterior-axillary line. The primary assistant held the 3D endoscope and stood on the left side of the lead surgeon. All surgical maneuvers were performed by visualizing the monitor through 3D glasses.

**Figure 3:**
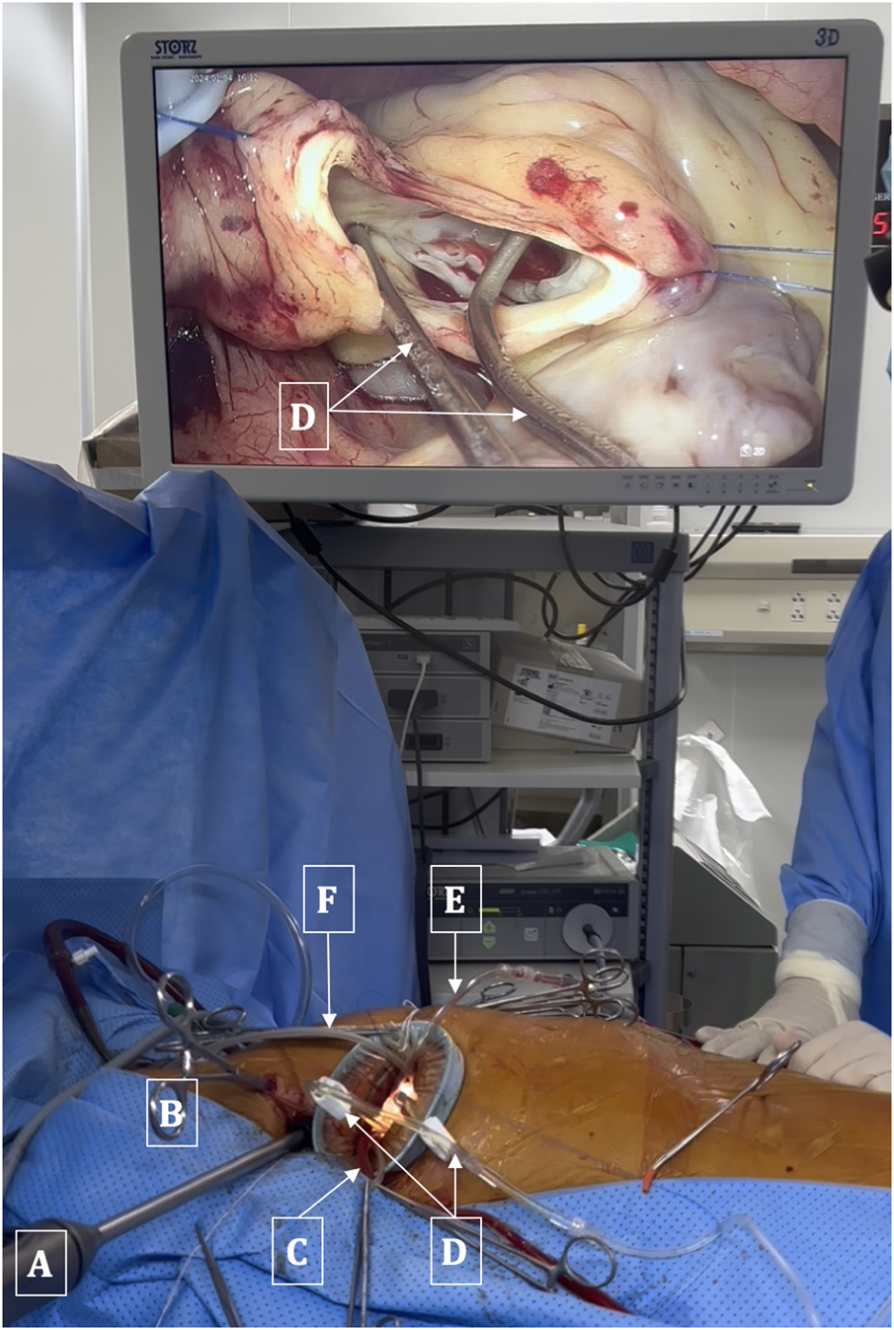
Arrangement of surgical instruments. **(A)** 3D Endoscopic camera. **(B)** Chitwood clamp. **(C)** Left venting line. **(D)** Dual cannulae for administration of cardioplegia solution into coronary ostia. **(E)** Aortic suction line. **(F)** CO_2_ Insufflation line.

The ascending aorta was cross-clamped with a transthoracic Chitwood clamp through the 2nd ICS at the anterior axillary line. Cardioplegia was delivered into the ascending aorta or directly into the coronary ostia (post-aortotomy in patients with aortic valve regurgitation). A suture was used to secure or tie around the cardioplegia needle and retract it towards the anterior chest wall to avoid interference between this needle and other surgical instruments. The left venting line was placed through the superior right pulmonary vein and passed through the main working port. Prior to clamping the aorta, in cases of aortic valve regurgitation, the left venting line was placed and three stitches were sutured into the aortic wall. These three stitches were used as retraction sutures to expose the aortic valve and the coronary ostia immediately after opening the aorta, thereby minimizing the time during which the myocardium was unprotected (Figure 3).

**Figure 4:**
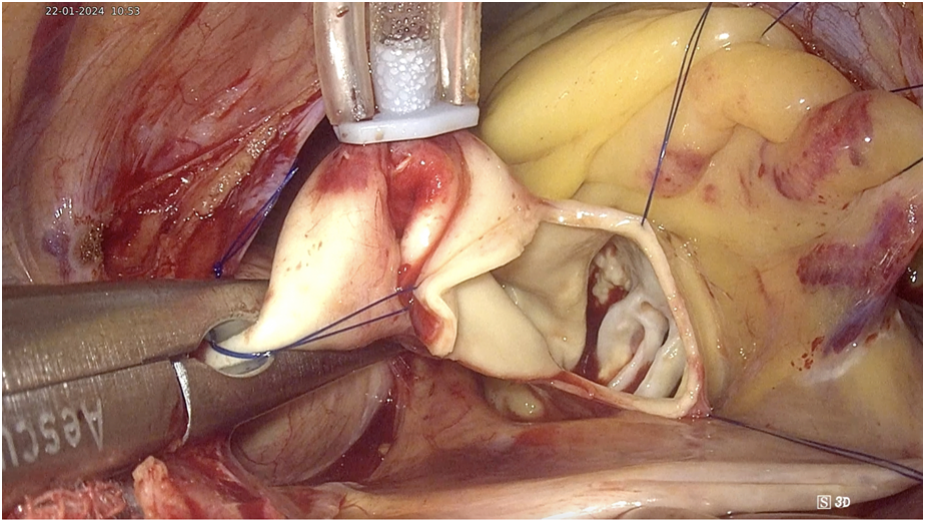
Exposure of aortic valve with retraction sutures.

The transverse aortotomy was typically made about 2 cm above the root of the right coronary artery. The aortic valve was exposed using three retraction sutures. Two sutures were used to retract the lower aortic wall towards the diaphragm and anterior chest wall, while one suture pulled the upper aortic wall towards the pericardium, thereby providing complete exposure of the aortic valve (Figure 4).

**Figure 5:**
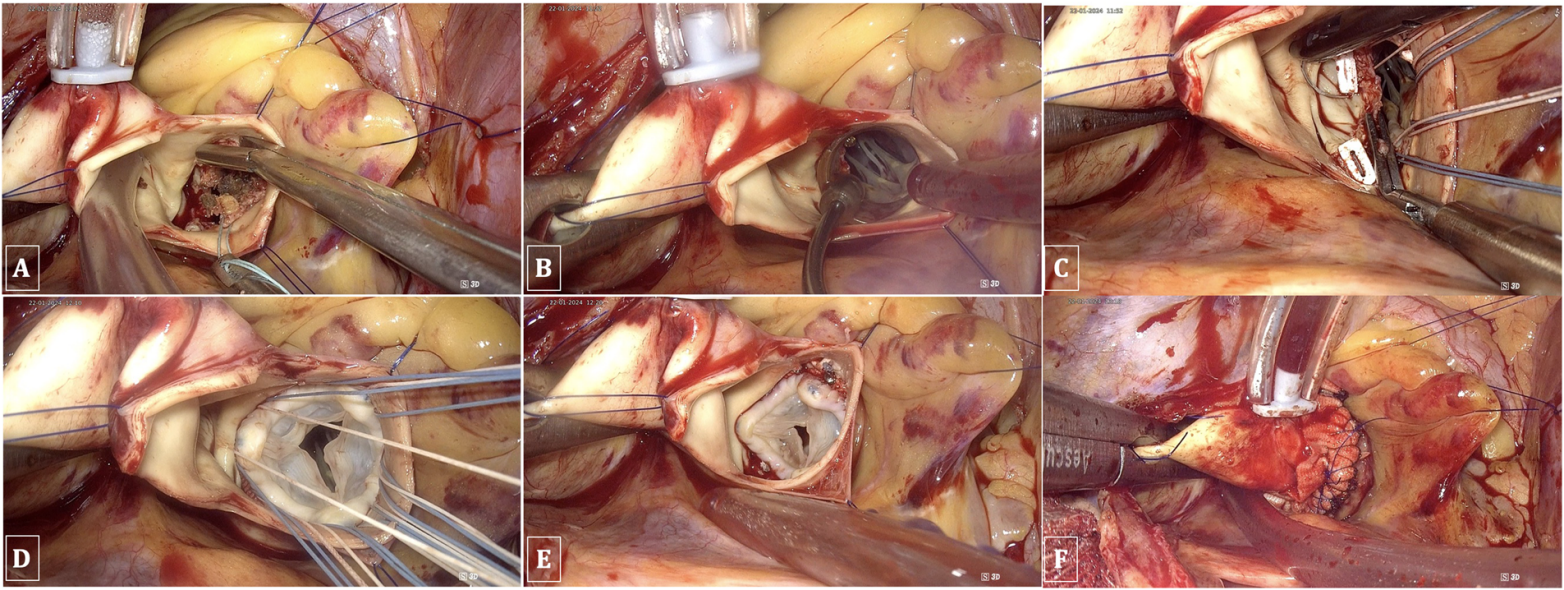
TEAVR. **(A)** Resection of the aortic valve and debridement of calcifications. **(B)** Evaluating the valve size. **(C)** Suturing the aortic valve annulus. **(D)** Implantation of a bioprosthetic aortic valve. **(E)** Securing and trimming sutures using the Cor-Knot device. **(F)** Double-layer closure of the aorta with reinforcement patches.

The native valve and calcifications of the leaflets and annulus were resected under the observation of a 3D endoscope to ensure thorough removal of all small fragments, thereby preventing any debris from falling into the left ventricle during the process. Interrupted sutures with pledgets were employed on the annulus in the following sequence: starting from the noncoronary annulus clockwise to the right coronary annulus, counterclockwise, and finally to the left coronary leaflet, ensuring that the sutures did not obscure the endoscopic view. The prosthesis was implanted in the usual manner, and the valve stitches were secured with a Cor-Knot device (LSI Solutions, NY, USA). Finally, the aorta was closed using a double 4/0 polypropylene running suture. All these procedures were performed under complete endoscopic vision (Figure 5).

## 3 Results

### 3.1 Patient characteristics

A cohort of 35 patients underwent TEAVR. The mean age of the patients was 58.7 ± 12.8 years, with 8 (22.9%) being female. The distribution of New York Heart Association (NYHA) functional classes was as follows: NYHA I in 6 patients (17.1%), NYHA II in 23 patients (65.7%), NYHA III in 5 patients (14.3%), and NYHA IV in 1 patient (2.9%).

Comorbidities included hypertension in 15 patients (42.9%), diabetes mellitus in 5 patients (14.3%), and chronic obstructive pulmonary disease (COPD) in 1 patient (2.9%). No patients had a history of previous myocardial infarction, renal insufficiency, stroke, or coronary artery disease. Atrial fibrillation was observed in 3 patients (8.6%), and no cases of peripheral vascular disease were reported.

Echocardiographic assessments indicated a mean left ventricular ejection fraction (LVEF) of 53.1 ± 29.2%. Specifically, LVEF was greater than 45% in 11 patients (31.4%), between 35% and 45% in 20 patients (57.1%), and between 25% and 35% in 4 patients (11.4%). The mean left ventricular end-diastolic dimension (LVEDD) was 53.5 ± 10.9 mm, and the mean pulmonary artery systolic pressure was 40.7 ± 15.9 mmHg. Regarding aortic valve pathology, 12 patients (34.3%) had aortic valve stenosis (AS), 8 patients (22.9%) had aortic valve regurgitation (AR), and 15 patients (42.8%) had mixed AS and AR (Table 1).

**Table 1:** Preoperative baseline data.

Age (years), mean ± SD	58.7 ± 12.8
Female, *n* (%)	8 (22.9)
NYHA class	
NYHA I, *n* (%)	6 (17.1)
NYHA II, *n* (%)	23 (65.7)
NYHA III, *n* (%)	5 (14.3)
NYHA IV, *n* (%)	1 (2.9)
Comorbidities	
Hypertension, *n* (%)	15 (42.9)
Diabetes, *n* (%)	5 (14.3)
COPD, *n* (%)	1 (2.9)
Previous myocardial infarction, *n* (%)	0
Renal insufficiency, *n* (%)	0
Atrial fibrillation, *n* (%)	3 (8.6)
Stroke, *n* (%)	0
Coronary artery disease	0
Peripheral vascular disease	0
Echocardiographic details	
LVEF %, mean ± SD	53.1 ± 29.2
LVEF >45%, *n* (%)	11 (31.4)
LVEF 35%–45%, *n* (%)	20 (57.1)
LVEF 25%–35%, *n* (%)	4 (11.4)
LVEF <25%, *n* (%)	0
LVEDD mm, mean ± SD	53.5 ± 10.9
Pulmonary artery systolic pressure, mean ± SD	40.7 ± 15.9
Aortic valve stenosis (AS), *n* (%)	12 (34.3%)
Aortic valve regurgitation (AR), *n* (%)	8 (22.9%)
Mixed AS & AR, *n* (%)	15 (42.8%)

### 3.2 Intraoperative data

The median CPB time was 210 ± 43 min, while the median aortic cross-clamp time was 132 ± 41 min. Prosthetic valve types included mechanical prostheses in 11 patients (31.4%) and bioprostheses in 24 patients (68.6%). The sizes of the prosthetic valves were distributed as follows: 19 mm in 1 patient (2.9%), 21 mm in 17 patients (48.5%), 23 mm in 12 patients (34.3%), and 25 mm in 5 patients (14.3%). One patient (2.9%) required repair of a ventricular septal with a patch. There were no cases requiring sternotomy; all patients successfully underwent aortic valve replacement using a totally endoscopic approach (Table 2).

**Table 2:** Intraoperative patients variables.

CPB time (min), median (SD)	210 (43)
Cross-clamp time (min), median (SD)	132 (41)
Prosthesis type	
Mechanical, *n* (%)	11 (31.4)
Bioprothesis, *n* (%)	24 (68.6)
Prosthesis size	
19 mm	1 (2.9)
21 mm	17 (48.5%)
23 mm	12 (34.3%)
25 mm	5 (14.3%)
Ventricular septal repair	1 (2.9)
Conversion to full sternotomy	0

### 3.3 Postoperative results

The median duration of mechanical ventilation was 15 h, and the medianlength of stay in the ICU was 3 days. Prolonged mechanical ventilation (>48 h) was needed in 6 patients (17.1%). Reoperation for bleeding was required in 1 patient (2.9%), and the medianvolume of red blood cell transfusion was 575 ml. There were no occurrences of minor stroke, though reversible neurologic injury was reported in 1 patient (2.9%).

New renal failure necessitating hemodialysis occurred in 2 patients (5.7%). No patients experienced vascular complications or required permanent pacemaker implantation. New-onset atrial fibrillation was observed in 1 patient (2.9%), and no cases of paravalvular leakage were identified. The early (30-day) mortality rate was observed in 2 patients (5.7%) (Table 3).

**Table 3:** Postoperative results.

Mechanical ventilation time (h), median (SD)	15 (132)
ICU stay (days), median (SD)	3 (3.9)
Prolonged mechanical ventilation (>48 h), *n* (%)	6 (17.1)
Bleeding requiring reoperation, *n* (%)	1 (2.9)
Red blood cell transfusion (ml), median (SD)	575 (628)
Stroke (minor), *n* (%)	0
Reversible neurologic injury, *n* (%)	1 (2.9)
New renal failure requiring hemodialysis, *n* (%)	2 (5.7)
Vascular complication, *n* (%)	0
Permanent pace maker implantation, *n* (%)	0
New-onset AF, *n* (%)	1 (2.9)
Paravalvular leakage, *n* (%)	0
Early (30-day) mortality, *n* (%)	2 (5.7)

## 4 Discussion

In recent years, the trend toward minimally invasive cardiac surgery has gained increasing popularity, even in developing countries such as Vietnam. Globally, numerous studies have been conducted on minimally invasive aortic valve replacement through small incisions, such as ministernotomy, right anterior minithoracotomy, right parasternal approach, and transverse sternotomy (2). However, comprehensive studies focusing on TEAVR remain relatively uncommon.In this study, we assess the feasibility and initial outcomes of TEAVR performed via a single working port at the 4th ICS on the right anterior axillary line. Aortic valve replacement via a right thoracotomy was first described in 1993 by Rao P.N. and Kumar A.S. (3). The minimally invasive approach through partial sternotomy, introduced in 1996 by Cosgrove D.M. and Sabik J.F. (4), has since gained widespread acceptance within the surgical community. Various modified approaches have subsequently been developed, including transverse sternotomy, superior sternotomy, inferior sternotomy, reverse “Z” sternotomy, and “I” or “J” sternotomy. The primary objectives of these methods are to minimize surgical trauma while ensuring both safety and efficacy (5).

Currently, the most commonly utilized minimally invasive techniques for aortic valve replacement are right anterior thoracotomy and upper hemisternotomy. Although partial sternotomy avoids the necessity of a full sternotomy, it can still compromise sternal stability and carries a risk of postoperative sternal infection. The right anterior thoracotomy approach primarily relies on direct visualization, which does not fully leverage the advantages of endoscopic surgery. Furthermore, in cases where the aorta is shifted to the left, direct access to the aortic valve becomes challenging. The limited anterior-posterior chest cavity space further restricts the maneuverability of surgical instruments.

Recently, with the advancement of technology, particularly in 3D endoscopy, several studies have investigated the application of completely endoscopic techniques in aortic valve replacement. In Wenda Gu's study, two working ports were utilized: The endoscopic camera was placed at the 4th ICS and the main working port was situated between the parasternal line and the midclavicular line of the right 3rd ICS (6). Daniele Zoni's study employed a 3- to 4-cm right mini-thoracotomy in the 2nd or 3rd ICS at the midclavicular line (7). Soh Hosoba's study implemented a 3-port technique: a 3- to 4-cm main port without rib spreading at the 4th ICS, a 10-mm 3D endoscopic port through the 4th ICS on the right mid-axillary line, and a 5-mm left-hand port at the 2nd or 3rd ICS on the right anterior axillary line (8).

**Figure 6:**
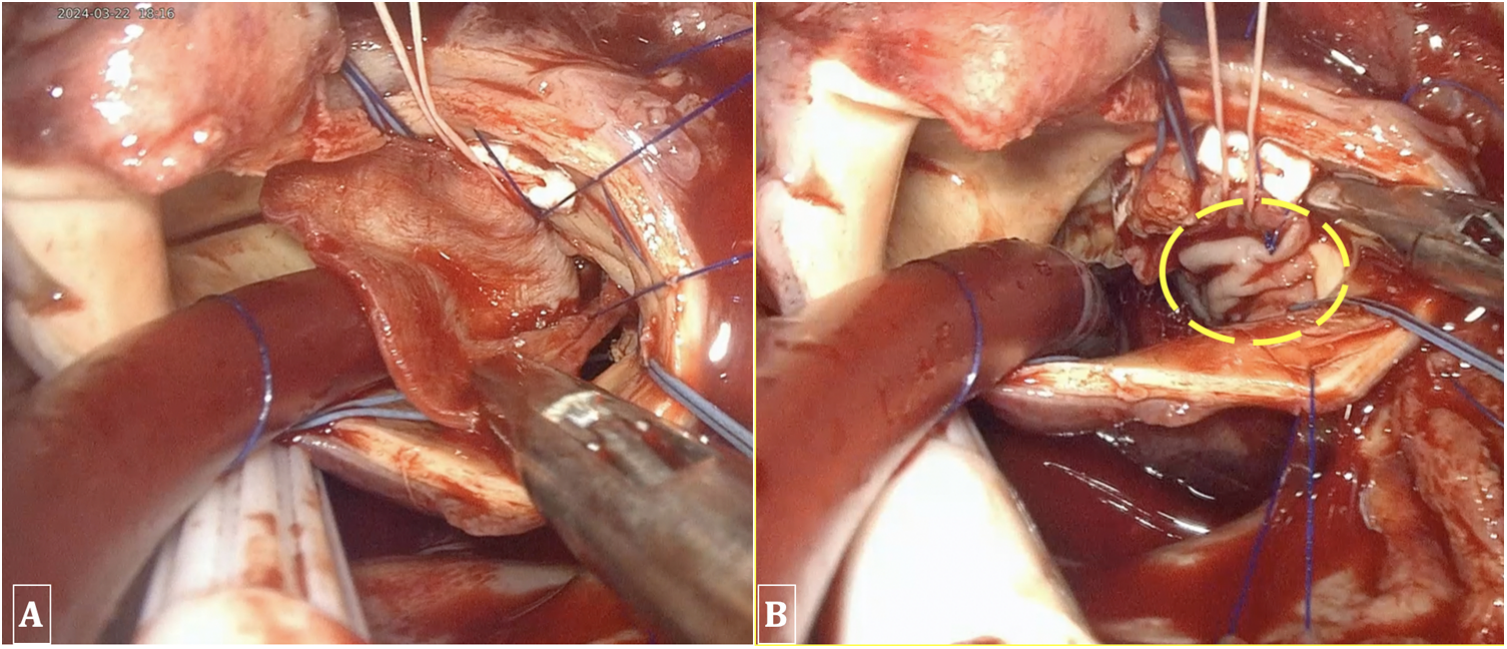
Performing ventricular septal repair and annuloplasty using a bovine patch. **(A)** A 2 × 3 cm bovine patch utilized for the repair of the ventricular septum. **(B)** The patch is sutured into the interventricular septum and the aortic annulus.

Our study utilized a totally endoscopic approach with a 3D camera positioned in the 3rd ICS along the mid-axillary or anterior axillary line, with all surgical instruments introduced through a single working port at the 4th ICS. This setup was deemed the most straightforward due to our prior experience and familiarity with the operative field in endoscopic mitral valve surgery. The main working port at the 4th ICS permits optimal access to the aortic valve across a variety of anatomical configurations. The working space for endoscopic instruments is also expanded and more advantageous due to the broader intercostal spaces located more posteriorly. Positioning the endoscopic camera in the 3rd ICS allows direct visualization of the aortic valve and coronary ostia. In our initial cohort of 35 patients, we determined that aortic valve replacement via a single working port at the 4th ICS utilizing 3D endoscopy is entirely feasible. This technique was successfully applied to patients with aortic regurgitation and aortic stenosis, including those with severe calcification of the valve leaflets and annulus. Additionally, we encountered a patient with severe aortic regurgitation due to infective endocarditis. Following the excision of the valve leaflets and the debridement of infected tissue that had extended to the annulus at the membranous septum, a communication between the left and right ventricles was identified. In this case, it was necessary to repair the ventricular septum with a bovin patch and replace the aortic valve with a mechanical prosthesis (Figure 6). This particular case had the longest aortic cross-clamp and CPB times in our study, at 243 min and 339 min, respectively. This patient, along with the remaining 34 cases, underwent successful aortic valve replacement via endoscopy without necessitating the extension of the thoracic incision or conversion to full sternotomy. However, the extended time required to manage such lesions increases the surgical risk. Therefore, thorough evaluation is essential for patients with infective endocarditis, particularly those suspected of having annular abscesses, to exclude them from candidacy for endoscopic surgery.

### 4.1 Myocardial protection

In this study, myocardial protection was achieved by cross-clamping the ascending aorta using a Chitwood clamp inserted through the 2nd ICS at the anterior axillary line. A long aorta needle was introduced through the main working port to administer antegrade cardioplegia solution via the aortic root, or directly into the coronary ostia following aortotomy. In cases of aortic valve regurgitation necessitating the placement of two cannulas for cardioplegia directly into the coronary ostia, the 3D endoscopic camera provided superior and more precise visualization of the coronary ostia compared to traditional sternotomy. The cardioplegia needle was retained for de-airing after declamping. To prevent the cardioplegia needle from interfering with the endoscopic instruments during valve replacement, a retraction suture was utilized to pull this needle towards the anterior chest wall, thereby avoiding obstruction of the surgical instruments.

### 4.2 Exposure and replacement of the aortic valve

The aortotomy was performed approximately 2 cm above the root of the right coronary artery. The aortic valve was exposed using three retraction sutures, which pulled the aortic wall towards the anterior chest wall, diaphragm, and pericardium.

In the context of Vietnam, where sutureless aortic valves are not widely available, we employed traditional prosthetic valves. The resection of the native valve and debridement of calcified tissue were facilitated by the endoscopic setup and the strategic placement of surgical instruments. However, when suturing the aortic annulus, the use of interrupted stitches introduced through the main working port posed a challenge, as the previous sutures could obstruct the endoscopic view when placing subsequent ones. To address this, we adopted a systematic suturing sequence: beginning with the noncoronary annulus in a clockwise direction, followed by the right coronary annulus in a counterclockwise direction, and finally the left coronary annulus (either clockwise or counterclockwise). This sequence ensured unobstructed visualization of each suture. On average, we utilized approximately 12–15 sutures for aortic valves sized 19, 21, or 23 mm, and 15–18 sutures for the 23 or 25 mm valves. The process of knot-tying in endoscopic aortic valve replacement, using a standard knot pusher, is relatively time-consuming due to the limited operative field compared to endoscopic mitral valve surgery. To streamline this process, we employed automated fastener devices or self-suturing devices, such as Cor-Knot, which significantly reduced both the aortic cross-clamp and CPB times.

### 4.3 Early outcomes

There were two mortalities in our study. The first case involved a patient who experienced postoperative bleeding necessitating re-exploration. We performed the reoperation at the sixth hour, reinforcing the aortic sutures via endoscopy. However, the patient succumbed on the third day due to low cardiac output syndrome. The second case involved a patient who developed multiple cerebral infarctions, identified on the second postoperative day. The patient's condition progressively deteriorated due to subsequent infection and renal failure requiring dialysis, resulting in death on the twentiethday. Both patients were among the first ten cases operated on, and the complications of postoperative bleeding and irreversible cerebral injury (possibly due to inadequate de-airing), contributed to the mortality rate of 5.7% among the 35 patients. The observed mortality rate of 5.7% in our cohort is indeed higher than the mortality rates reported in the STS and SCTS databases for isolated AVR. This discrepancy may reflect the initial challenges faced during the early adoption of the TEAVR technique, including the steep learning curve and the management of complex cases that were not fully anticipated preoperatively. It is important to compare these outcomes with those of TAVR (Transcatheter Aortic Valve Replacement) and mini-sternotomy approaches, which have been shown to offer lower mortality rates in similar patient populations. While TEAVR has the potential to become a viable alternative to these established techniques, our findings suggest that further refinement of the procedure and increased surgical experience are necessary to reduce mortality and improve overall outcomes. Future studies should aim to directly compare TEAVR with TAVR and mini-sternotomy to better define its role in the treatment of isolated aortic valve disease.

The CPB time and aortic cross-clamp time in our study were 210 ± 43 min and 132 ± 41 min, respectively, which are relatively long compared to other reports. This can be attributed to the fact that the majority of patients in our study (77.1%) presented with aortic stenosis (both isolated and mixed), often accompanied by severe calcification, which extended the time required for valve excision and annular debridement. Additionally, one patient required repair of a ventricular septal injury, resulting in CPB and cross-clamp times of 339 min and 243 min, respectively. Furthermore, during the initial cases, our limited experience in valve exposure and annular suturing contributed to the prolonged operative times. However, these durations are anticipated to decrease with increased surgical experience and the integration of specialized endoscopic instruments, such as advanced tools for valve decalcification and automated aortic suturing devices. Moreover, the implementation of sutureless aortic valves may further contribute to a reduction in operative times.

Despite these challenges, the TEAVR technique offers several advantages. This approach is less invasive compared to traditional sternotomy, which may lead to reduced surgical trauma, faster recovery times, and earlier mobilization. However, while these benefits are not evident in our current study outcomes, they may be realized in future research with a larger patient cohort and the availability of more advanced technical resources. Additionally, the use of a 3D endoscopic system enhances visualization, enabling surgeons to perform aortic valve replacements through smaller incisions, thereby reducing surgical invasiveness, while also ensuring greater precision in complex cases, particularly in those with appropriate clinical indications.

## 5 Limitations

However, there are notable limitations to our study. As this is a newly introduced surgical technique, it requires a steep learning curve, which may have contributed to the observed high mortality rate. It is crucial to carefully select appropriate patients to mitigate risks. Furthermore, the study lacks a control group with sternotomy, limiting direct comparisons. Long-term follow-up is necessary to fully understand the outcomes, and larger sample sizes are needed to comprehensively assess both the safety and feasibility of the TEAVR technique.

## 6 Conclusion

TEAVR via the 4th ICS along the anterior axillary line has demonstrated its feasibility as a less invasive alternative to the traditional sternotomy for aortic valve replacement. This technique is particularly advantageous in cases not requiring extensive aortic root enlargement or the management of complex aortic annular abscesses. While the initial operative times were extended, reflecting the learning curve associated with this procedure, it is anticipated that these times will decrease with accumulated surgical experience. The results of this study suggest that, with further refinement and optimization, TEAVR holds significant potential to supplant conventional sternotomy in select patient populations. Nonetheless, additional research and larger-scale studies are warranted to further validate these findings, optimize procedural efficiency, and evaluate long-term outcomes to fully establish the role of TEAVR in clinical practice.

## Data Availability

The original contributions presented in the study are included in the article/Supplementary Material, further inquiries can be directed to the corresponding author.

## References

[1] Glauber M Farneti A Solinas M Karimov J. Aortic valve replacement through a right minithoracotomy. Multimed Man Cardiothorac Surg. (2006) 2006(1110):mmcts.2005.001826. 24413458 10.1510/mmcts.2005.001826

[2] Glauber M Ferrarini M Miceli A. Minimally invasive aortic valve surgery: state of the art and future directions. Ann Cardiothorac Surg. (2015) 4(1):26–32. 25694973 10.3978/j.issn.2225-319X.2015.01.01 PMC4311160

[3] Rao PN Kumar AS. Aortic valve replacement through right thoracotomy. Tex Heart Inst J. (1993) 20(4):307–8. 8298332 PMC325118

[4] Cosgrove DM 3rd Sabik JF. Minimally invasive approach for aortic valve operations. Ann Thorac Surg. (1996) 62(2):596–7. 10.1016/0003-4975(96)00418-3 8694642

[5] Salenger R Gammie JS Collins JA. Minimally invasive aortic valve replacement. J Card Surg. (2016) 31(1):38–50. 10.1111/jocs.12652 26466846

[6] Gu W Zhou K Wang Z Zang X Guo H Gao Q Totally endoscopic aortic valve replacement: techniques and early results. Front Cardiovasc Med. (2022) 9:1106845. 10.3389/fcvm.2022.1106845 36698939 PMC9868623

[7] Zoni D Cresce GD Hinna-Danesi T Benvegnù L Poddi S Gallo M Endoscopic aortic valve surgery in isolated and concomitant procedures. Interdiscip Cardiovasc Thorac Surg. (2023) 36(6). 37326963 10.1093/icvts/ivad101 PMC10371047

[8] Hosoba S Ito T Mori M Kato R Kajiyama K Maeda S Endoscopic aortic valve replacement: initial outcomes of isolated and concomitant surgery. Ann Thorac Surg. (2023) 116(4):744–9. 10.1016/j.athoracsur.2023.04.045 37276923

